# Production stability and biomass quality in microalgal cultivation – Contribution of community dynamics

**DOI:** 10.1002/elsc.201900015

**Published:** 2019-03-27

**Authors:** Martin Olofsson, Elin Lindehoff, Catherine Legrand

**Affiliations:** ^1^ Centre for Ecology and Evolution in Microbial Model Systems (EEMiS) Department of Biology and Environmental Science (BoM) Linnæus University Kalmar Sweden

**Keywords:** biomass composition, flue gas, microalgal cultivation, multispecies communities, production stability

## Abstract

The prospect of using constructed communities of microalgae in algal cultivation was confirmed in this study. Three different algal communities, constructed of diatoms (*Diatom*), green algae (*Green*), and cyanobacteria (*Cyano*), each mixed with a natural community of microalgae were cultivated in batch and semi‐continuous mode and fed CO_2_ or cement flue gas (12–15% CO_2_). *Diatom* had the highest growth rate but *Green* had the highest yield. Changes in the community composition occurred throughout the experiment. Green algae were the most competitive group, while filamentous cyanobacteria were outcompeted. Euglenoids, recruited from scarce species in the natural community became a large part of the biomass in semi‐steady state in all communities. High temporal and yield stability were demonstrated in all communities during semi‐steady state. Valuable products (lipids, proteins, and carbohydrates) comprised 61.5 ± 5% of ash‐free biomass and were similar for the three communities with lipids ranging 14–26% of dry mass (DM), proteins (15–28% DM) and carbohydrates (9–23% DM). Our results indicate that culture functions (stability, biomass quality) were maintained while dynamic changes occurred in community composition. We propose that a multispecies community approach can aid sustainability in microalgal cultivation, through complementary use of resources and higher culture stability.

**Abbreviations**
Chla: chlorophyll *a*
DM: dry mass
FG: cement flue gas
NC: natural microalgal community
PBR: photobioreactor
TC: total carbohydrates
TL: total lipids
TP: total proteins

## 1 INTRODUCTION

Microalgal cultivation and production coupled to CO_2_ bioremediation of industry flue gas is appealing to environmental managers, general public, and business entrepreneurs. The efficiency and sustainability of microalgal CO_2_ mitigation is intensively debated 1, 2, 3 and remains to be demonstrated in large scale. For algal production, using readily available industry flue gas as a CO_2_ source could facilitate outdoor mass cultivation of algal biomass to become sustainable, possibly combined with the utilization of other waste streams for fertilizing algal cultures.

Microalgal products range from bulk chemicals (lipids, proteins, and carbohydrates) to more specific metabolites such as pigments, fatty acids, polysaccharides, polyesters, amino acids, and peptides extracted and refined from the algal biomass or from cells 4, 5, 6, 7. There is also a broad spectrum of potential industries in the race for algal products and product development, ranging from biofuel, feed, food to biomaterial, and pharmaceutical industry 8, 9, 10. If algal product development could be coupled to bioremediation through recycling of waste streams such as wastewater and exhaust gases, there may be both economic and environmental benefits contributing to a bio‐based economy.

Using flue gas as a CO_2_ source exposes algal cells to other compounds in the flue gas besides CO_2_. Cement flue gas also contains considerable amounts of toxicants, including SO_x_ and NO_x_ gases 11. These compounds can act alone or together to form a cocktail with synergistic effects potentially harming cells or impeding growth, mainly due to pH stress. However, Olofsson et al. 12 demonstrated that cement flue gas was an appropriate CO_2_ source for microalgae with the same or higher biomass production attained compared to industrial grade CO_2_.

Outdoor large‐scale algal cultivation necessitates a stable ecosystem resilient to rapid changes due to both environmental factors and imposed growth conditions. It has been implied that multispecies communities or mixed cultures may be more suitable for large‐scale applications, both in terms of sturdiness and stability 13, 14, 15, 16, 17, but rarely outperform the most productive species in the community 13, 15. Using multispecies communities in crop farmlands was shown potentially to increase biomass productivity through stable ecosystems and niche differentiation [13, 14, 18,], which was suggested to apply also for microbial systems 9, 15, 16, 17, 18, 19, 20. Natural brackish microalgal communities, dominated by diatoms, showed high quality of biomass and higher productivity than a Baltic Sea diatom monoculture at maximum yield 12. Freshwater microalgal polycultures with high functional group richness showed higher algal lipid content with increasing species richness 19, 20, 21, 22.

**PRACTICAL APPLICATION**

Our study “Production stability and biomass quality in microalgal cultivation – Contribution of community dynamics” is one of the few addressing the seasonality and constructed community approach in microalgal cultivation. We also show that constructed communities of brackish Baltic Sea microalgal communities performed equally in terms of production stability and biomass quality (high value products) regardless of community composition. The use of community approach adds knowledge and options for further use in biotechnological production system using microalgae for applications in sustainable feed supplements and biofuels. Multispecies community approach has the potential to increase stability and productivity, to simplify system management, and reduce the risk of contamination or collapse. To mimic the natural succession takes advantage of local adaptations to light and temperature and increase the potential of maximum biomass production.

All community approaches to cultivation remain unproven at a large scale for microalgae. The use of microbial multispecies consortia in biotechnology, while not new, is uncommon in algal biological solutions compared to bacterial applications. An outdoor production system is subject to seasonal changes, such as light and temperature, which has an impact on algal biomass quantity and quality 23, 24. In developing a sustainable cultivation system, the use of natural algal communities would favor the stability of biomass productivity regarding adaptation to local and seasonal conditions. As biodiversity increases the productivity and stability of phytoplankton communities 16, 25, 26, natural algal communities should be less susceptible to pathogens, invasive species, and zooplankton grazing.

To approach whether the natural succession over the year with seasonally adapted species can aid in the stability of algal cultivation, we used constructed microalgal communities representative for seasonal dynamics in the brackish Baltic Sea. Cold‐water diatoms and dinoflagellates dominate the spring bloom, succeeded by blooms of filamentous cyanobacteria (phylum bacteria) in the summer 27, 28, 29. Dinoflagellates are also present in the plankton community in late summer and autumn. Green pigmented microalgae (Chlorophyceae, Euglenoids, Eustigmatophyceae) are common in the littoral zone at warm temperatures (late summer, early autumn). Diatoms, cyanobacteria, and green algae are good candidates for large‐scale biomass production of valuable products 29, 30, 31. The aim of this study was to compare different microalgal communities, constructed in way to reflect natural seasonal succession, and to test their performance in terms of stable biomass production, and valuable products under controlled conditions. Tolerance of the constructed communities to different sources of CO_2_ (CO_2_‐air mix and flue gas) was tested.

## 2 MATERIALS AND METHODS

### 2.1 Microalgal stock cultures and natural community

Microalgal strains were acquired from the Kalmar Algal Collection, the Finnish Environment Institute (SYKE), Bigelow–the National Center for Marine Algae and Microbiota (formerly CCMP), and Necton S.A. (Olhão, Portugal) (Supporting Information Table 1). All species were grown in batch in filtered (0.2 μm) Baltic seawater (salinity 7) supplied with Guillard's f/2 + Si medium and original f/2, respectively 32. Cultures were grown in 5 L glass flasks, gently bubbled with air at 15°C and irradiance 300–500 μmol photons s^−1^ m^−2^. Irradiance was measured with a digital scalar irradiance meter (Biospherical Instruments Inc).

The natural microalgal community (NC) was collected in the upper mixed layer, July 1, 2013, at Linnæus Microbial Observatory in the Baltic Sea, Southeast Sweden, (N 56°55.851, E 17°03.640). Zooplankton was removed from the water with a 100 μm nylon net. Seawater was kept cool and in the dark less than 12 hours prior to the experiments. The phytoplankton community was dominated by filamentous cyanobacteria (*Anabaena* sp., *Aphanizomenon* sp., *Nodularia spumigena*), the dinoflagellate *Dinophysis* sp. and chain‐forming diatoms, mainly *Skeletonema marinoi*. Lower abundances of other dinoflagellates, cryptophytes, and smaller green flagellates (2–20 μm) were also observed.

### 2.2 Constructed communities

To provide information on the stability of production of mixed algal communities in relation to seasonal dynamics, we developed three constructed microalgal communities (*Diatom, Green, Cyano*) using species isolated from the Baltic Sea. The constructed communities were composed of a set of three single species belonging to the same order/family inoculated in NC (Supporting Information Table 1). The chosen species are morphologically different and possible to distinguish from each other with light microscopy.

Single species and NC were inoculated to 25% of the total “pigmented” biomass (final concentration based on chlorophyll *a* (Chla). Each constructed community (*Diatom, Green, Cyano*) and treatments (CO_2_ and FG) were inoculated in triplicate tubular polystyrene photobioreactors (type PBR2 in 12). Final volume (4.5 L) was adjusted with filtered Baltic seawater (<0.2 μm) enriched with f/2 or f/2+Si. Light was supplied by metal‐halide lamps (250 W Osram) in a 16 h Light: 8 h Dark cycle at an irradiance of 300–500 μmol m^−2^ s^−1^, measured with a digital scalar irradiance meter (Biospherical Instruments Inc.). Aeration and gas (CO_2_‐ air mixture or FG) additions through air stone 0 to 20 mm of the cylinder bottom. In the three constructed communities, initial Chla concentrations (5.8–6.3 μg/L) and biovolume were comparable (131–170 × 10^−2^ mm^3^ L^−1^) (Supporting Information Table 1).

### 2.3 Culture operations

Replicate PBRs were continuously sparged with air during the experiment. Three times during the light period (08.00, 12.00, and 16.00), the cultures were fed either industrial grade CO_2_‐air mixture or cement flue gas (FG) for 1 min at a 1 L/min flow. Adding CO_2_ or flue gas decreased the pH‐value in the cultures by 1 to 1.5 units, but the pH‐value recovered gradually to initial levels (3–4 h) prior to subsequent addition. Collection and composition of cement flue gas (FG) are described in 12. As reference, CO_2_‐air mixture (industrial grade, 13.5% CO_2_) was obtained from AGA Gas AB.

### 2.4 Experimental set‐up

To determine basic growth rates and optimal production of the three constructed communities in nonlimiting conditions, the PBR2 were run in fed‐batch mode over 7–10 days. Inorganic nutrients were monitored during exponential growth (fed‐batch) and adjusted accordingly to f/2+Si levels to prevent limitation at high biomass (data not shown). Daily pH‐ (HI98128 hand pH‐meter, Hanna instruments) and Chla levels were measured. Start and endpoint samples of cell abundance and community composition were collected and fixed with Lugol's solution prior to microscopical identification as in 12. Samples for algal biomass dry mass (DM) were collected at start and end of batch. Biomass for chemical composition (lipids, proteins, and carbohydrates) were sampled at batch endpoint and frozen (–20°C) prior to analyses.

To test for the stability of the biomass productivity and its quality of the three constructed communities over time, the PBR2 were run in semi‐continuous mode 10 days after dilution of the fed‐batch cultures. Dilution began when Chla levels indicated a transition into stationary phase due to light limitation (Day 7 for *Diatom*, Day 10 for *Green* and *Cyano*). In all PBR2, 25–30% of the culture volume was withdrawn daily from the reactor, and replaced with an equal volume of fresh f/2+Si medium. Dilution rates were 0.30 d^−1^ (*Diatom*), 0.25 d^−1^ (*Green*), and 0.25 d^−1^ (*Cyano*). Nutrient levels were monitored and shown to be in excess (data not shown). Semi‐steady state mode was defined as when the biomass stabilized within 20% daily variation. Chlorophyll a, DM and pH‐ values were measured and analyzed daily. Biomass for chemical composition was taken at the end of semi‐continuous mode and stored in –20°C prior to analyses.

### 2.5 Microalgal biomass and community composition

Cell numbers and community composition of the dominant species were determined from fixed samples (Lugol's solution) using 10 mL or 100 μL sedimentation chambers 33. At least 300 cells were counted in each sample using an inverted light microscope (Olympus BX 50), resulting in an expected error of 5–10%. Constructed community species were visually identified and cells originating from the natural background community were identified to the genus level, when possible. Cell biovolume was calculated according to Olenina et al. 34 and multiplied with cell numbers or length of filaments (cyanobacteria) to express total biovolume of each species/community in the sample (mm^3^ L^−1^). Chla analysis was carried out by 95% ethanol extraction 35. The extract from 5–20 mL filtered (25 mm glass fibre PALL A/E) culture suspension was then measured in a Turner Trilogy fluorometer. Maximum specific growth rate (d^−1^) was determined by plotting the natural logarithm of the Chla series concentration and derived the value from the steepest slope of four consecutive data points (days 1–5 for *Diatom* and *Green*, days 3–7 for *Cyano*). For dry mass (DM), 25–100 mL of culture was filtered onto prewashed (Milli‐Q), dried and preweighed 45 mm glass fibre filters (Whatman GF/C). Cells were rinsed on filters with 10 mL Milli‐Q to remove excess salt and oven‐dried at 100°C until constant weight was recorded using a Mettler Toledo, College‐B B154 scale. The weight difference of dried filters with biomass and empty filters divided with filtered volume was given as mg L^−1^ DM. Productivity in semi‐steady state mode (mg L^−1^ d^−1^) was calculated as the average of daily productivity values based on DM for two consecutive days accounting for daily dilution factor. To illustrate the temporal and yield stability in semi‐steady state, a linear regression including 95% confidence interval was used based on daily productivity calculated from daily DM yield. Theoretical maximum production during semi‐steady state was calculated as in 12 times a dilution factor corresponding to 50% of the maximum growth rate of each constructed community, assuming no light, and nutrient limitation.

### 2.6 Biochemical composition

Aliquots of algal suspension (1L) were centrifuged for 20 min at 11,900 × *g* (Beckman, Avanti J‐25). Most of the supernatant was discarded and the pellet was redissolved in 20–50 mL of the supernatant left in the centrifugation bottle (400 mL), transferred to 50 mL Falcon tubes and further centrifuged at 2850 × g (Hettich Universal 16R). Pellets were stored in –20°C prior to vacuum drying in a Labconco bulk tray drier and a Scanvac Coolsafe until constant weight. For respective analysis of total protein and total carbohydrate, 2 × 5 mg (duplicates) of biomass were then transferred to clean glass tubes. The remaining biomass (30–50 mg) was used to quantify total lipids.

### 2.7 Total lipids

Lipids were extracted according to Bligh & Dyer 36 with slight modification. Chloroform:MeOH 1:2 (vol:vol) was used as solvent and added to the dried biomass. Sonication ruptured algal cell walls using a Sonics Vibra‐cell (VCX 130) for 2 min at 50% of maximum amplitude. The solution was then centrifuged (10 min, 2850 × *g*, Hettich Universal 16R) and the supernatant collected in a new tube. New solvent was added to the centrifuged pellet and the extraction procedure (including sonication) was repeated three times. Milli‐Q was added to the collected supernatant at the final proportions of Chloroform: MeOH: H_2_O 2:2:1 and vortexed until homogenous, followed by centrifugation (10 min, 2850 × *g*, Hettich Universal 16R) for separation. The aqueous‐methanol layer was removed and the lipid‐chloroform layer was transferred to preweighed aluminium baking cups allowing the chloroform to evaporate in a fume hood for 24 h. The aluminium cups were then dried at 60°C until constant weight quantified gravimetrically on a Mettler Toledo, College‐B B154 scale.

### 2.8 Total proteins

Duplicates of 5 mg dried algal biomass were mixed with 2 mL 1 M NaOH and incubated for 60 min in a 95°C water batch. Subsequently, samples were centrifuged for 10 min at 2850 × *g* (Hettich Universal 16R). Hundred micro liters of the supernatant was transferred to a glass tube and used together with a ready‐made assay (Bio‐Rad DC Protein assay kit II) according to Lowry et al. 37. Samples prepared with the protein assay was measured in a spectrophotometer (WWR UV‐1600 PC) at 750 nm and compared to a bovine serum albumin (included in the assay) standard curve ranging 0.2–1.0 mg L^−1^.

### 2.9 Total carbohydrates

The Phenol‐H_2_SO_4_ method 38 was used to extract the carbohydrates from the algal biomass. Dried biomass was resuspended in 5 mL 1 M H_2_SO_4_ and incubated for 60 min in a water bath at 90°C. Samples cooled down to room temperature and were centrifuged for 10 min at 2850 × *g* (Hettich Universal 16R). Hundred micro liters of the supernatant was transferred to a glass vial and mixed with 1 mL of phenol (5% w/v) and 3 mL of H_2_SO_4_ (72 % wt %). Samples together with a standard series (Sigma D‐(+)‐glucose, 0.2–1.0 mg L^−1^) were incubated in a water bath for 5 min (90°C). Absorbance at 490 nm was read in a spectrophotometer (WWR UV‐1600 PC) and concentrations were derived from the function of the standard curve.

### 2.10 Statistics

**Figure 1:**
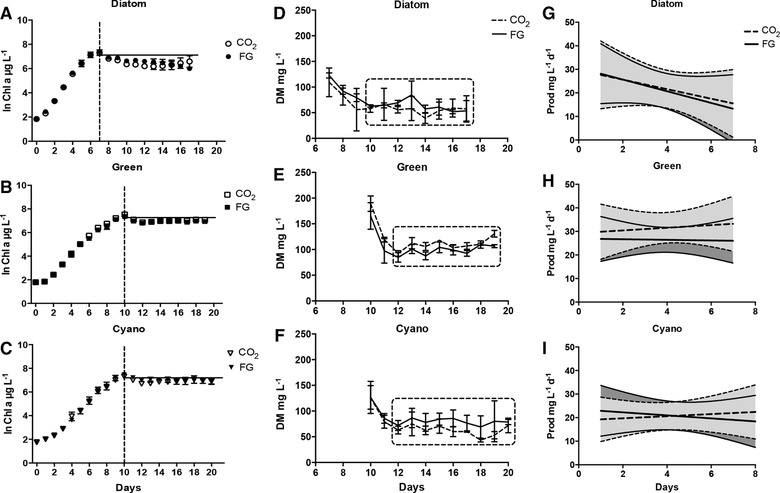
Three constructed communities; *Diatom, Green*, and *Cyano* were fed CO_2_ or cement flue gas (FG). Mean values ± SD, *n *= 3 are shown in (A–C) natural logarithm of chlorophyll *a* (Chla) concentrations during batch and semi‐continuous growth. The dashed vertical lines indicate the start of semi‐continuous mode. The solid horizontal lines indicate average dilution rates during semi‐continuous mode (*Diatom* (0.30 d^−1^), *Green* (0.25 d^−1^), and *Cyano* (0.25 d^−1^)). (D–F) Yield at semi‐continuous mode when fed CO_2_ (dashed line) or flue gas (FG) (solid line). Cultures reached semi‐steady state (dashed box) at day 10–17 (*Diatom*), day 12–19 (*Green*), and day 12–20 (*Cyano*). (G–I) Temporal and yield stability during semi‐steady state when fed CO_2_ (dashed line) or flue gas (FG) (solid line), regression line with 95% CI (shaded area)

Statistical analyses were performed using GraphPad Prism (version 6.0d for Mac OS X, GraphPad Software, La Jolla, California, USA). Endpoint values of Chla, growth rate (based on Chla), DM at batch, average DM during semi‐steady state and average productivity at semi‐continuous mode/steady state were analyzed by two‐way ANOVAs. Yield stability was analyzed using a linear regression (Figure 1 I) and the differences of the slopes were tested using ANCOVA. For all statistics, the *p *< 0.05 level of significance was chosen. Multiple comparisons (Tukey's post‐hoc test) of ANOVA were reported with multiplicity adjusted *p* values to account for multiple comparisons.

## 3 RESULTS

### 3.1 Growth, biomass yield, and production

In this study, the source of CO_2_ (CO_2_‐air mixture or FG) had no effect on any of the measured variables, (two‐way ANOVAs, simple effects within communities *p *> 0.05). However, observed effects of microalgal community (*Diatom, Green, Cyano*) were found (two‐way ANOVAs, main community effects *p *< 0.05) and will be explained in this section.

The growth rate of *Diatom* was significantly higher compared to the two other communities (two‐way ANOVA Community: *p *< 0.0001,; *Diatom* vs. *Green p *< 0.0001; *Diatom* vs. *Cyano p *< 0.0001) (Table 1, Figure 1C). *Green* and *Cyano* had similar growth rates (Table 1 . The communities reached late exponential phase at day 7 (*Diatom*) and day 10 (*Green* and *Cyano*) with similar Chla maximum yields (Figure 1C). No significant differences of batch Chla endpoint values among communities were found (two‐way ANOVA *p *> 0.05). In contrast, dry weight (DM) yield, ranging 109–189 mg L^−1^ (Table 1, was significantly different among communities (Two‐way ANOVA Community:, *p *< 0.01). *Green* had significantly higher yield (189 and 165 mg L^−1^ for CO_2_ and FG, respectively) compared to both *Diatom* (109 and 123 mg L^−1^ for CO_2_ and FG, respectively) and *Cyano* (127 mg L^−1^ regardless of treatment) (two‐way ANOVA, *p *< 0.01 and *p *< 0.01, respectively).

**Table 1:** Growth and yield parameters from batch and semi‐continuous mode in microalgal constructed communities fed CO_2_ or flue gas (FG)

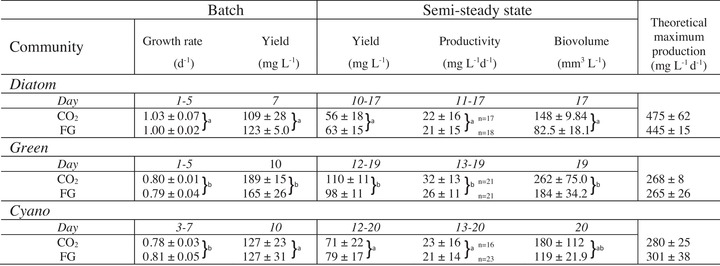

Biomass productivity during semi‐steady state is calculated from yield values (DW, mean ± SD, *n*) shown in Figure 3. Different letters indicate statistically significant differences (*p *< 0.05) of multiple comparisons (two‐way ANOVA, Tukey's test, main community effect). Any letter in common indicates no significance. Error range is given in SD (*n *= 3).

All communities reached semi‐steady state 2–3 days after start of dilution (Figure 1F). *Green* showed the lowest variation in biomass among communities during semi‐steady state.

Community effects (Two‐way ANOVA Community: *p *< 0.0001) irrespective of CO_2_ treatment were also observed at semi‐steady state where the average DM yield was significantly higher for *Green* (98–110 mg L^−1^), compared to both *Cyano* (65–79 mg L^−1^) and *Diatom* (56–63 mg L^−1^) (two‐way ANOVA, *p *< 0.001 and *p *< 0.0001, respectively) (Table 1).

*Diatom* (406–524 mg L^−1^ d^−1^) had a higher theoretical maximum production, compared to both *Green* (236–287 mg L^−1^ d^−1^) and *Cyano* (252–339 mg L^−1^ d^−1^) due to higher growth rate (Table 1). In terms of total biovolume *Green* was significantly higher than *Diatom* (two‐way ANOVA, *p <* 0.05) regardless of treatment.

The linear regression with 95% CI illustrates both temporal and yield stability of community production during semi‐steady state, and revealed high temporal stability in all communities (Figure 1I).

Neither of the regression lines of all the communities significantly deviated from 0 (Simple Regressions *p* > 0.1). Comparing slopes of regression lines revealed no significant difference among communities and treatments (ANCOVA, *p *> 0.05). The width of the 95% confidence intervals of the slopes (shaded area in Figure 1I), 5.1 (*Cyano*), 5.9 (*Green*), and 7.8 (*Diatom*), indicated a higher variation in yield stability for *Diatom*. Productivity ranged 21–32 mg L^−1^ d^−1^ for all communities and a significant effect of community on average daily productivity at semi‐steady state (Table 1 was found (two‐way ANOVA, *p *< 0.05). *Green* had significantly higher productivity compared to both *Diatom* and *Cyano* (two‐way ANOVA, *p <* 0.05 and *p *< 0.05, respectively).

### 3.2 Community composition

**Figure 2:**
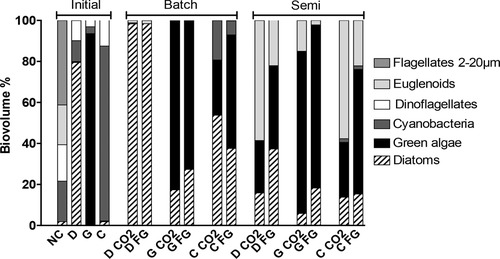
Composition of the initial natural community (NC) and the different constructed communities *Diatom* (D), *Green* (G), and *Cyano* (C) at start, end of batch and end of semi‐continuous mode. Cultures were fed CO_2_ or flue gas (FG). Mean values, *n *= 3

In the initial NC, dinoflagellates (43%), cyanobacteria (48%), diatoms (4%), and green algae (5%) were the dominant groups in terms of biovolume (Figure 2). In the constructed communities, the target groups (diatoms, green algae and cyanobacteria) made up at least 80% of the pigmented biomass.

During batch mode in *Diatom*, diatoms outcompeted any other groups representing up to 98% of the biovolume. Community composition changed drastically during semi‐continuous mode as the proportions of diatoms decreased to 16% (CO_2_) and 37% (FG) (Figure 2). Diatoms were replaced by Euglenoids (22–59%) and green algae (25–41%). In *Green*, green algae decreased from 90% to 75% of the biovolume in batch mode to the benefit of diatoms (20%). *Green* composition did not change during semi‐continuous mode and Euglenoids replaced part of the diatoms (5–10%). In *Cyano*, filamentous cyanobacteria decreased from 80% to less than 20% of the biovolume, replaced equally by green algae and diatoms. After semi‐continuous mode, less than 2% of filamentous cyanobacteria were left while diatoms (20%), green algae (20–50%) and Euglenoids (20–50%) made up the rest of the biomass. Euglenoids were the only group affected by the source of CO_2_ with less contribution to the biomass in FG.

### 3.3 Quality of biomass composition for different microalgal communities

**Figure 3:**
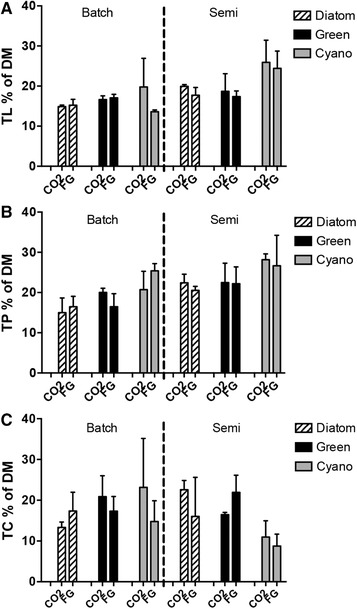
Bulk chemicals (A) Total lipids (TL), (B) total protein (TP), and (C) total carbohydrates (TC) in % of dry mass (DM) for *Diatom*, *Green*, and *Cyano* communities in batch and semi‐continuous mode fed CO_2_ or flue gas (FG). Mean values ± SD, *n *= 3

Valuable products (lipids, proteins, and carbohydrates) comprised of 61.5 ± 5% of the biomass dry mass (DM) ash free. The biochemical composition of all three communities was comparable during batch mode despite changes in community composition (Figures 2 and 3). However, significant differences of multiple comparisons were found within total lipids (TL) (two‐way ANOVA Community; *p *< 0.001) and total proteins (TP) (two‐way ANOVA Community; *p *< 0.001). Protein content in *Cyano* was significantly higher than *Diatom* (two‐way ANOVA Community; *p *< 0.05). In semi‐continuous mode, TL ranged 14–26% DM (Figure 3). Concomitant with the change in community composition in *Cyano* between batch and semi‐continuous mode, lipid content was significantly higher at the end of the experiment (Figures 2 and 3)(two‐way ANOVA Community; *p *< 0.001). Further, *Cyano* TL was significantly higher than in *Diatom* and *Green* (two‐way ANOVA Community; *p *< 0.05). In general, TP (15–28% DM) and total carbohydrates (TC) (9–23% DM) of the three communities did not show any significant differences although there is a trend that *Cyano* TC decreased from batch to semi‐steady state and were lower than *Diatom* and *Green* (Figure 3).

## 4 DISCUSSION

Environmental fluctuations, contamination by pathogens and grazers often undermine industrial‐scale algae cultivation. Ongoing research, using fundamental ecological principles, particularly the stability and resilience of diverse microalgal communities, as a powerful approach toward the sustainability of industrial‐scale algal cultivation has received increasing attention 12, 37, 38, 39. Moreover, industrial flue gas can be an inexpensive source of CO_2_ used in the cultivation process 12. The present study reports production performance, stability, and biomass quality of three constructed microalgal communities fed cement flue gas. Among the constructed communities tested, *Diatom* had the highest specific growth rate, thereby the highest theoretical maximum production assuming nonlimiting light and resources conditions at a 0.5 d^−1^ dilution rate. However, *Green* had the highest yield in both batch and semi‐steady state. High temporal and yield stability during semi‐steady state was showed in all communities, demonstrating the potential of using diverse constructed communities in algal production.

Recently, we reported that natural and diverse diatom communities had similar biomass production and quality (lipids, proteins, carbohydrates) as diatom monocultures 12. The overlapping traits of the different diatom species (*Skeletonema* spp. and *Chaetoceros* spp.) may explain the lack of differences in yield and products between a diverse community and a monoculture. To explore further the diversity‐production relationship in microalgae, we used major algal taxonomic groups that differ in their physiology and biochemical properties, thus present functional variations. In the current study, initial constructed communities were composed of 70% of species belonging to the same functional group (diatoms, green algae, filamentous cyanobacteria) and a diverse NC. One could therefore argue that functional redundancy may have occurred within a community as functional diversity more than species richness was found by several to enhance productivity and stability 16, 22, 40. Godwin et al. 41 also showed that polycultures outperformed monocultures in terms of multi‐functionality (biocrude production and more efficient use of N and P). However, the NC provided a rich mix of species, e.g. functional richness, increasing the resource‐use complementarity in the communities as revealed by the success of diatoms in all batch treatments. Euglenoids, exclusively recruited from the NC, had an advantage over other groups in all communities during semi‐steady state, showing the importance of scarce species (e.g. low abundance) 42. The dominance of native green algae and Euglenoids also confirms previous findings concerning biomass production, where native species were superior to commercial strains 9. This also implies enhanced stability by the NC to the culture. Euglenoids are common in nutrient rich and eutrophied waters 43, 44 and were highly competitive in the CO_2_ treatment but considerably less so in the FG treatment where Euglenoids were replaced by green algae. Flue gas did affect negatively the biovolume of Euglenoids compared to CO_2_, although cement FG has been shown to be a suitable source of CO_2_ for Baltic microalgae 12. The presence of an inhibitory compound in the cement FG that targets specifically Euglenoids cannot be dismissed.

During semi‐continuous mode, the natural algal succession within the PBR system changed both community composition and dynamics for all constructed communities while biomass production was stable within a community. Hence, production stability may be a result from contribution of microalgal groups with complementary characteristics 20, 39 as three different phylogenetic groups (diatoms, green algae, Euglenoids) were co‐occurring in the PBR. Nitrogen‐fixing filamentous cyanobacteria, usually dominant during summer in calm and warm brackish waters with low N:P ratio, were rapidly outcompeted under any of the culturing conditions in this study (abundant N supply, continuous aeration with pulses of CO_2_/FG). This result indicates that potential biomass toxicity due to filamentous cyanobacteria can be ruled out in brackish cultivation systems.

Studies of biomass composition for multispecies communities are scarce. Cea‐Barcia et al. 45 reported carbohydrates ranging 12–57% volatile solids (VS) for natural communities dominated by freshwater Chlorophytes grown in domestic secondary effluents. Stockenreiter et al. 21, 22 showed increased lipid accumulation with both increased species diversity (up to four different species) and functional diversity. In the present study, algal biomass quality was equivalent among different microalgal communities and between treatments, although with some intriguing differences. Lipid content in *Cyano* increased during semi‐steady state compared to batch endpoint, reflecting the change in community composition with an increased dominance of green algae and Euglenoids together with a minor diatom contribution. While *Cyano* turned very similar to both *Diatom* and *Green* during steady state in terms of species composition, its biochemical quality was different. Community composition can also be shaped by chemical interactions between microalgae and cyanobacteria 46. The minute presence of filamentous cyanobacteria in the community could still produce bioactive substances and influence the function of the co‐existing groups. Following the reasoning of natural succession in outdoor algal cultivation systems, our result suggests that a multispecies community dominated by diatoms (spring) will produce mainly proteins and carbohydrates. Communities by green algae and other flagellates (e.g. Euglenoids) are likely to be competitive during summer‐autumn and can provide a stable biomass quality production.

The presence of microalgal groups with various traits creates a niche differentiation in light utilization possibly resulting in higher biomass production and lipid yield as found by Stockenreiter et al. 22 and proposed by Smith et al. 47, 48.

Diverse cultures are suggested to perform similar or better than single‐species cultures due to higher resilience, less susceptibility to contamination, and more efficient usage of resources 12, 16, 17, 19, 22, 25, 26. Thus, using a multi‐species community approach (natural or constructed) can aid sustainability, meaning less labor, and cost for the efforts of maintaining single‐species cultures. Some taxa will naturally be more competitive than others under certain conditions. In the present study, green algae and Euglenoids (CO_2_ treatment) rose to dominance, indicating a selection effect where the conditions benefit one or a few number of species 49. Thus, both complementary effect and selection effect combined could contribute to stability of microalgal cultivation. However, the effects of diversity on productivity and stability are not clearly predictable and may vary with the combination of species more than the actual biodiversity itself 16. Constructing or assembling suitable algal communities will depend on the required function (e.g. biomass or a specific compound) and local environmental conditions. Different phylogenetic or functional groups of microalgae can operate different niches (this study), but a trait‐based approach where different light and nutrient utilization, temperature, and pH optima, tolerance to shear stress, and biochemical composition should also be considered 16, 17, 39. In the conditions of our study, natural succession and selection resulted in functionally diverse algal groups with complementary light capturing capacities (groups with different pigmentation) and temperature optimums. An alternative would be to create a selective system for a specific function (biomass or lipids) and grow a natural or constructed community where competition will select suitable communities and maintain innate function. Nonetheless, our results showed that community composition changes over time and species may alternate but function is maintained (production stability with little change in biomass quality). Thus, the diverse system with the constructed communities in the present study implied culture resilience with the possibility that invading species either could contribute or do little harm to the community functions as suggested by Kazamia et al. 22.

There is a wealth of microalgal diversity with approximately 40,000 identified species 50. Considering the mutual relationships within ecosystems that have evolved over billions of years, nature itself may bring sustainability to algal technology. Sustainability of commercial‐scale cultivation is more likely to be achieved when the use of multi‐species communities can provide the annual stability through complementary effects and culture resilience. Our results are promising and outdoor scale‐up trials (1600 L) of natural community cultures confirm the notion of production stability and biomass quality 51. Adding concepts of seasonal succession in combination with crop rotation 40 can only increase the possibilities. However, algal product development must allow flexibility, as target products can vary with dominant microalgal groups throughout the growth season. Extended research should be directed into large‐scale demonstration of natural succession and crop rotation of microalgae. In a further notice to bridge the gap between R&D and commercialization, regional, and national long‐term agricultural policies ought to include algal cultivation into future strategies 52.

As a step toward reduced carbon footprint, CementaHeidelberg AB Degerhamn, Öland, southeast Sweden, works closely with Linnaeus University and SMA Mineral AB. The academia–industry collaboration is presently evaluating the possibility of using microalgal cultivation for CO_2_ mitigation of emitted flue gas. The tolerance and suitability of the flue gas released by Cementa AB, Degerhamn was previously investigated on production and biomass quality of selected microalgal strains and a diatom dominated NC 12. By extending this work to constructed communities, representative of seasonality, and natural succession, we demonstrated the broader potential for large‐scale algal cultivation with cement flue gas as CO_2_ source. No differences in bulk algal production were found between two gas sources, CO_2_ and FG, in terms of performance. Taking into account the results from this and our previous study, cement flue gas released by Cementa AB proved to be a nontoxic suitable CO_2_ source for microalgal cultivation.

## 5 CONCLUDING REMARKS

The approach of using constructed communities proved very useful in understanding the potential of using multispecies and natural assemblages in algal cultivation and product development. We demonstrated that a stable bulk production could be achieved using communities with different functional algal groups adapted to local environmental conditions, and relevant to the seasonal succession in the Baltic Sea. Our strategy revealed that while community composition is highly dynamic during cultivation, the complexity of the community ensured the stability of the production of bulk chemicals. Although stability was found for bulk chemicals, considering fine high value compounds, algal product development should apply a biorefinery concept to extract and refine a variety of compounds but exerted with great flexibility since different community compositions may yield different high value target compounds. A commercialization would likely involve a large corporate network targeting several markets. Further demonstration of the microalgal community approach at annual scaled‐up conditions should be a priority. The aim could be to increase R&D incrementally to commercial full‐scale algal cultivation integrated with other industry branches in coastal locations providing, for example CO_2_ from flue gas and nutrients from waste streams.

## CONFLICT OF INTEREST

The authors have declared no conflict of interest.

## Supporting information

Supporting Information
