# Supplementary material for: Production stability and biomass quality in microalgal cultivation – Contribution of community dynamics
Source: Eng Life Sci. 2019 Mar 27;19(5):330–40. doi: 10.1002/elsc.201900015 (PMC6999223; doi:10.1002/elsc.201900015)
Supplement: Supplementary file 1 — Supporting Information [file ELSC-19-330-s001.pdf]

Table S1. Contribution of different algal monocultures and natural phytoplankton community (NC) to the constructed communities: *Diatom*, *Green* and *Cyano*. Strains were obtained from different algal collections and a commercial strain (Necton c.s). Stock monocultures were diluted prior to mixing with NC. Start chlorophyll *a* (Chla) concentrations and biovolumes apply for each community fed CO_2 or industrial flue gas (FG). Biovolume was calculated based on cell biovolume (Olenina et al. 2006) and cell numbers. Mean values (n = 3) ± SD.

Community	Community composition	Strain	Stock Chla (µg L$^-$)	Dilution factor	Chla (µg L$^-$)	Biovolume (10^{-2} mm^3 L^{-1})	Biovolume (%)
Diatom	*Skeletonema marinoi*	SMTV1	316	240		29.8±1	23
	Phaeodactylum tricornutum	CCMP2928	727	560		33.7±2.4	26
	Chaetoceros wighamii	CWTVC1	256	200		33.6±12.6	26
	NC		1.8	2		33.7	26
	Start concentrations					131	100
	CO2				6.3±0.5		
	FG				6.2±0.1		
Green	*Tetraselmis* sp.	KAC21	129	100		63.7±17.3	30
	Dunaliella tertiolecta	CCMP1302	167	130		41.8±6.4	20
	Nannochloropsis oculata	Necton c.s.	220	170		72.9±11.4	34
	NC		1.8	2		33.7	16
	Start concentrations					165	100
	CO2				6.0±0.2		
	FG				5.9±0.1		
Cyano	*Aphanizomenon* sp.	KAC15	139	110		81.3±9.2	48
	Anabaena lemmermani	KAC16	664	510		19.5±8.1	12
	Nodularia spumigena	KAC7	274	210		34.8±32	21
	NC		1.8	2		33.7	20
	Start concentrations					170	100
	CO2				5.8±0.2		
	FG				6.1±0.2		
